# An isomorphous replacement method for efficient *de novo* phasing for serial femtosecond crystallography

**DOI:** 10.1038/srep14017

**Published:** 2015-09-11

**Authors:** Keitaro Yamashita, Dongqing Pan, Tomohiko Okuda, Michihiro Sugahara, Atsushi Kodan, Tomohiro Yamaguchi, Tomohiro Murai, Keiko Gomi, Naoki Kajiyama, Eiichi Mizohata, Mamoru Suzuki, Eriko Nango, Kensuke Tono, Yasumasa Joti, Takashi Kameshima, Jaehyun Park, Changyong Song, Takaki Hatsui, Makina Yabashi, So Iwata, Hiroaki Kato, Hideo Ago, Masaki Yamamoto, Toru Nakatsu

**Affiliations:** 1 RIKEN SPring-8 Center, Sayo, 679-5148, Japan; 2 Department of Structural Biology, Graduate School of Pharmaceutical Sciences, Kyoto University, Sakyo, 606-8501, Japan; 3 Institute for Integrated Cell-Material Sciences, Kyoto University, Sakyo, 606-8501, Japan; 4 Research and Development Division, Kikkoman Corporation, Noda, 278-0037, Japan; 5 Department of Applied Chemistry, Graduate School of Engineering, Osaka University, Suita, 565-0871, Japan; 6 Research Center for Structural and Functional Proteomics, Institute for Protein Research, Osaka University, Suita, 565-0871, Japan; 7 Japan Synchrotron Radiation Research Institute, Sayo, 679-5148, Japan; 8 Department of Cell Biology, Graduate School of Medicine, Kyoto University, Sakyo, 606-8501, Japan

## Abstract

Serial femtosecond crystallography (SFX) with X-ray free electron lasers (XFELs) holds great potential for structure determination of challenging proteins that are not amenable to producing large well diffracting crystals. Efficient *de novo* phasing methods are highly demanding and as such most SFX structures have been determined by molecular replacement methods. Here we employed single isomorphous replacement with anomalous scattering (SIRAS) for phasing and demonstrate successful application to SFX *de novo* phasing. Only about 20,000 patterns in total were needed for SIRAS phasing while single wavelength anomalous dispersion (SAD) phasing was unsuccessful with more than 80,000 patterns of derivative crystals. We employed high energy X-rays from SACLA (12.6 keV) to take advantage of the large anomalous enhancement near the *L*_III_ absorption edge of Hg, which is one of the most widely used heavy atoms for phasing in conventional protein crystallography. Hard XFEL is of benefit for *de novo* phasing in the use of routinely used heavy atoms and high resolution data collection.

Intense femtosecond X-ray pulses from XFELs offer new opportunities for protein crystallography. Serial femtosecond crystallography (SFX) has provided molecular structures from micron-sized protein crystals at ambient temperature^1^,^2^,^3^,^4^. With the increasing enthusiasm to apply this approach to a variety of proteins and complexes, great effort has been focused on the *de novo* structure determination methods^5^,^6^,^7^. Effective experimental phasing methods for SFX are essential to facilitate the realization of potential for XFEL-based protein crystallography for small protein crystals that are unsuitable for synchrotron beamlines.

Phase determination is the central problem in protein crystallography. Single wavelength anomalous dispersion (SAD) is now the most commonly used experimental phasing method^8^. After the successful application to macromolecular crystallography (MX) using synchrotron radiation, it has recently been extended to SFX. Barends *et al*. recently reported *de novo* phasing by the SAD method using SFX data of the tetragonal lysozyme crystals, collected at LCLS (Linac Coherent Light Source, U.S.)^7^. About 60,000 single pulse diffraction patterns were used to obtain an automatically traceable map. Strong anomalous contributions from two Gd ions (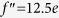 at 8.5 keV) and the high symmetry of the crystals (the point group 422) were beneficial in the study.

For SAD phasing, highly accurate intensity measurements are essential as this method utilizes weak anomalous differences (≲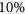 contribution to 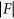). On the other hand, single isomorphous replacement with anomalous scattering (SIRAS) utilizes larger isomorphous differences (≳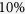 to 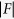) in addition to the anomalous signals. Although good isomorphism between native and derivative crystals is essential, this combination leads to the improved phasing reliability, which is more tolerant to intensity fluctuations compared with the SAD method. Here we apply SIRAS to SFX to assess its potential as a more efficient *de novo* method for phasing.

Heavy atoms, such as Se, Pt, Au, or Hg with absorption edges near 12.4 keV, have been frequently used to acquire phases using synchrotron radiation (SR). The SPring-8 Angstrom Compact free-electron Laser (SACLA) provides femtosecond X-ray pulses at such high photon energy to allow high resolution *de novo* structure determinations^9^. Whilst Selenomethionine (SeMet) labelling has been frequently used for recombinant proteins, Hg has been the most successful choice for isomorphous replacements^10^. Mercury typically binds covalently to cysteine residues and successful derivatization of microcrystals can be established by prescreening dissolving them and analyzing by mass spectrometry. This is beneficial because the beam time at XFEL sources is scarce and the huge amount of material is required to collect high quality data for establishing the presence of heavy atom.

We applied the SIRAS phasing method to obtain the structure of luciferin-regenerating enzyme (LRE)^11^. The LRE crystals belong to the space group *P*2_1_2_1_2_1_ and show one Hg site per asymmetric unit, composed of 308 amino acid residues (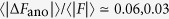 at full and half occupancy, respectively). In this SIRAS phasing, we used ~20,000 indexed patterns with 10,000 each for derivative and native crystals. A notable reduction in the number of diffraction patterns required for successful phasing of SFX data by SIRAS is achieved compared to that needed for SAD phasing. SIRAS is thus an efficient *de novo* phasing method for SFX studies.

## Results

### SIRAS phasing

SFX experiments on the LRE crystals were performed at BL3 of SACLA. For the native crystals, 133,958 images were collected. Of these, 26,238 images (20%) were preselected based on diffraction intensity and 10,792 patterns (41%) were indexed (see Methods). The Hg-derivative crystals were prepared by soaking and diffraction data were collected at a wavelength of 0.984 Å (12.6 keV). Out of 583,291 collected images, we selected 298,061 (51%), from which 85,747 patterns (29%) were indexed.

Diffraction patterns were processed using the CrystFEL software suite^12^. For native crystals, Monte-Carlo integration yielded the mean SFX multiplicity of 222 at 1.5 Å resolution, when all indexed patterns were used. For Hg-derivative crystals, the mean SFX (anomalous) multiplicity was 908 at 1.6 Å resolution over all indexed patterns. We tried phasing by SAD method using all Hg-derivative data but this was unsuccessful.

**Figure 1:**
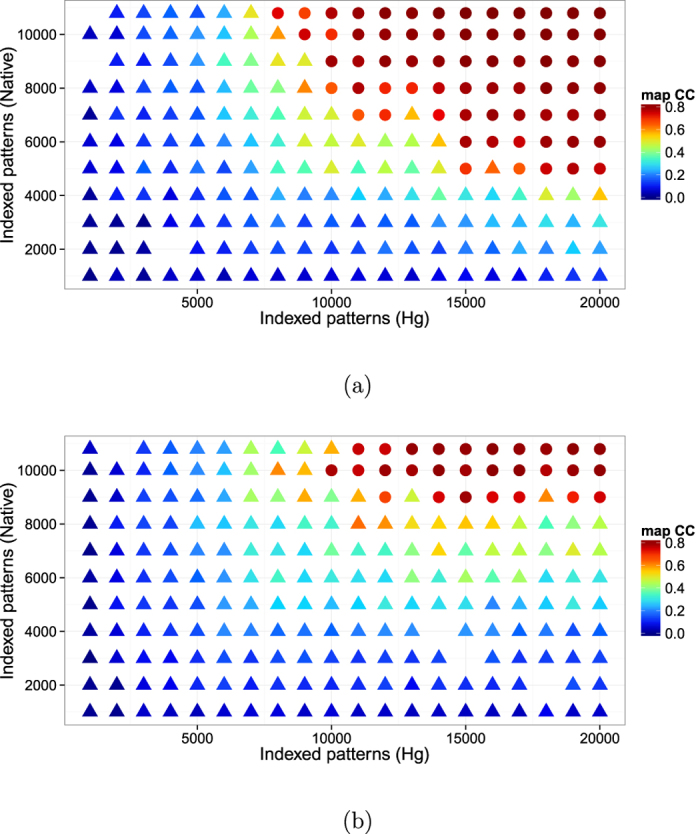
Phase quality (map CC) as functions of the numbers of the native and the derivative indexed patterns. (**a**) SIRAS case. (**b**) SIR case. The success and failure of phasing are represented as circular and triangular symbols, respectively. The CC of electron density maps (defined in the main text) is indicated by colors. Some data points are missing because SHELXE failed to trace map. The figure was prepared using R^36^ with ggplot2 package^37^.

We have tested various combinations of numbers of native and derivative patterns for SIRAS phasing (Fig. 1a). Using 10,792 native patterns, we found around 10,000 derivative patterns are sufficient for SIRAS phase determination with the mean multiplicity of 106. The data collection and refinement statistics are summarized in Table 1. Discrepancy in the unit cell parameters between the native LRE and the derivative was less than 0.2% and 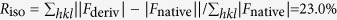 (calculated with Scaleit^13^).

**Figure 2:**
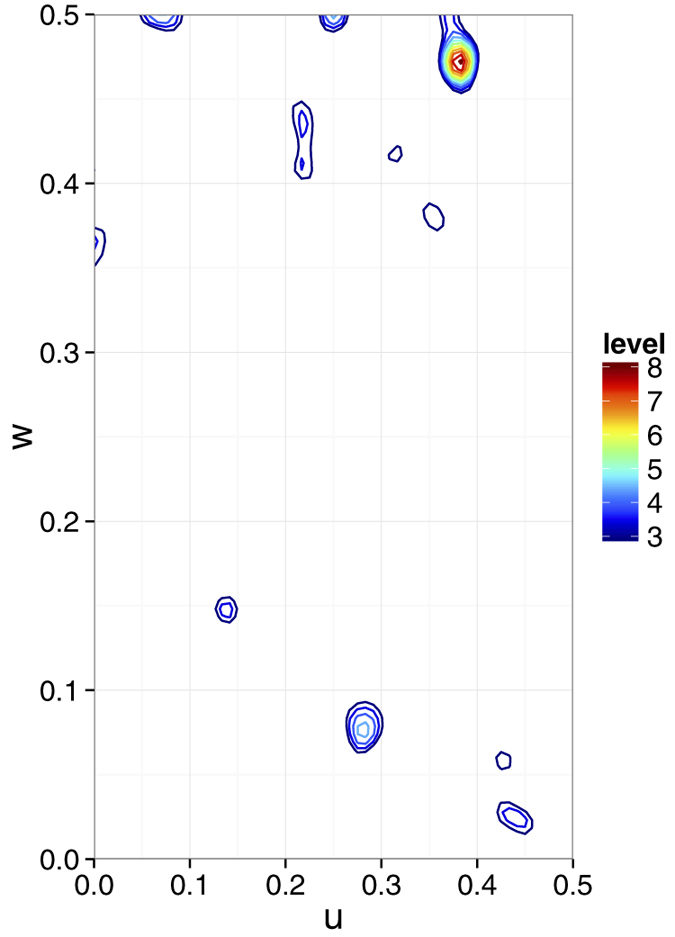
Isomorphous difference Patterson map. The *v* = 1/2 Harker section of the isomorphous difference 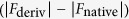 Patterson map calculated using indexed patterns from 10,792 native and 10,000 Hg derivative LRE crystals. The Patterson map was calculated using CCTBX functionality^38^. This figure was prepared using R^36^ with ggplot2 package^37^.

**Figure 3:**
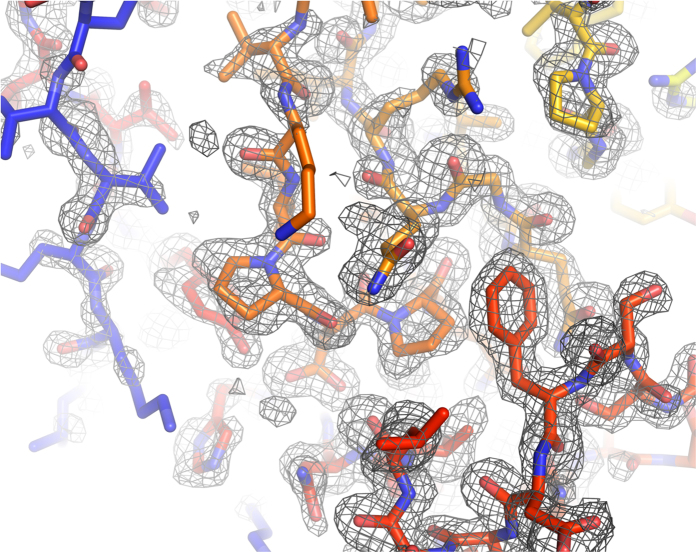
The SIRAS electron density map produced by SHELXE with a refined model of LRE. 10,000 and 10,792 patterns of derivative and native crystals were used for the calculation. The electron density map is contoured at 1.0*σ*. The figure was prepared using PyMOL^39^.

**Figure 4:**
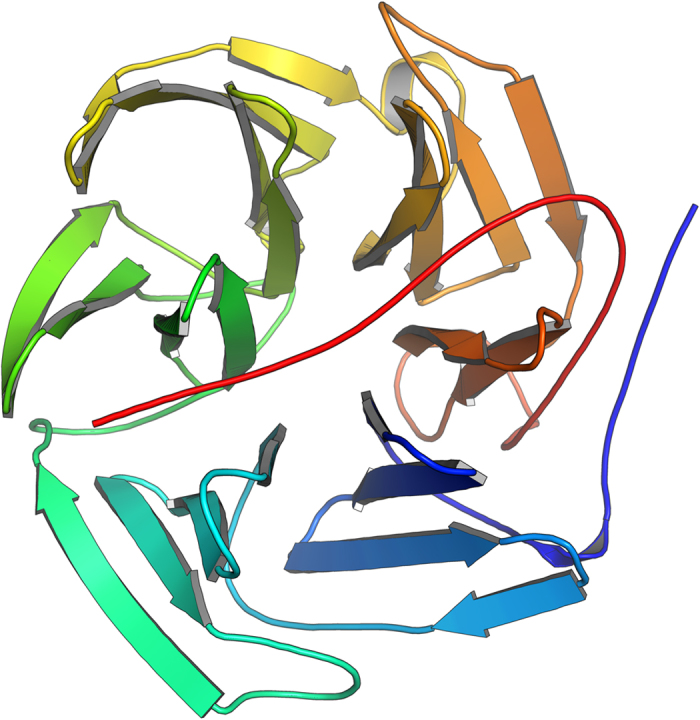
The atomic structure of LRE determined by SIRAS method. The model was refined against the SFX native data. The figure was prepared using PyMOL^39^.

Searches for the heavy atom substructure, phasing calculations and phase improvement were performed with the SHELX C, D, and E programs^14^. The position of one Hg atom per asymmetric unit was identified with SHELXD using the combination of isomorphous and anomalous differences with CC_all_ of 11.89% and CC_weak_ of 9.42%. An isomorphous difference Patterson map also showed a significant peak corresponding to the position of the Hg atom at 8.1*σ* (Fig. 2). Using this heavy atom position, phasing and phase improvement calculations were then carried out with SHELXE. The SHELXE procedure was repeated three times with 20 local cycles of density modification and poly-alanine auto-tracing. When the correct hand was used, the mean FOM of 0.615 was obtained, and 197 residues were modeled with CC = 27%. With the inverted hand, the mean FOM was 0.453 and 45 residues were modeled with CC = 6%. The electron density map for the correct hand was readily interpretable (Fig. 3). Automatic model building was then performed with ARP/wARP^15^ with REFMAC5^16^. 304 residues out of 308 were modeled with satisfactory accuracy (*R*_work_ = 22.3% and *R*_free_ = 27.6%). After a few cycles of manual model rebuilding using Coot^17^ and automated refinement using phenix.refine^18^, the refinement converged with residuals *R*_work_ = 18.5%% and *R*_free_ = 23.2% (Fig. 4). The model was also refined against the derivative data (*R*_work_ of 20.2% and *R*_free_ of 23.5%). In the anomalous difference Fourier map, two Hg sites with peak heights of 19.3*σ* and 3.52*σ* were identified near the Cys residue with occupancies of 0.66 and 0.16, respectively. The stereochemical qualities of the final refined model were analyzed using MolProbity in PHENIX^19^. The refinement statistics are summarized in Table 1.

### Number of indexed patterns and phase quality.

To study the relationship between the number of indexed patterns and the quality of *de novo* phasing, we repeated the SIRAS phasing protocol using different numbers of native and derivative patterns (Fig. 1a). As a phase quality indicator, we used the correlation coefficient (CC) between the experimental electron density map given by SHELXE and the *F*_c_ map calculated using the final refined structure mentioned above. When the CC value was greater than 0.65, the trial was judged a success. The minimum numbers of diffraction patterns required to locate the Hg site correctly were 1,000 for native and 2,000 for the derivative crystals (SFX multiplicity of 21; Supplementary Fig. 1a). For the phasing calculation, however, more than 5,000 native patterns and 8,000 derivative patterns were required but in total at least 18,000 patterns were needed for successful phasing.

The same protocol was repeated for SIR phasing *i.e*. without the use of the anomalous signals (Fig. 1b). This investigation showed SIR phasing requires more patterns than the SIRAS case, which ascertains that the anomalous signal contributes to the SIRAS phasing. For a successful phasing with SIR, 9,000 or more patterns collected from native crystals and 10,000 or more derivative patterns (at least 20,000 patterns in total) were required. When the anomalous signal was utilized (in the SIRAS case), the required number of native patterns was reduced if more derivative patterns were used. For heavy atom location, SIR resulted in a better correlation between calculated and observed substructure structure amplitudes, but SIRAS achieved a better phase accuracy after phase improvement (Supplementary Fig. 1).

**Figure 5:**
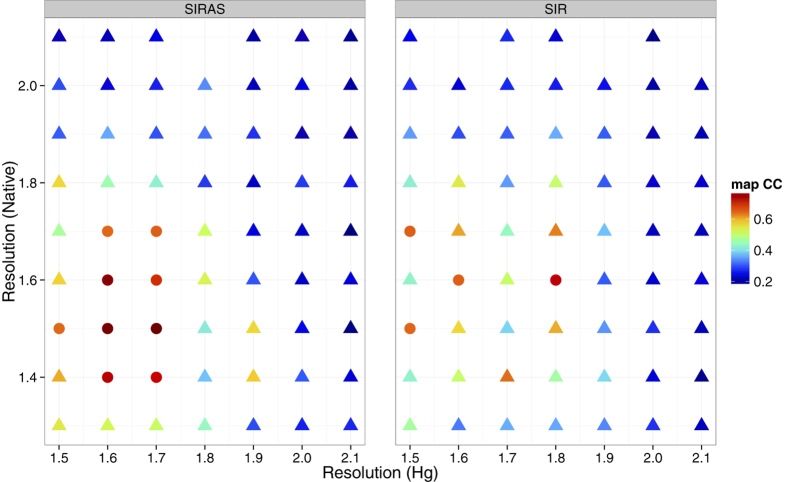
Effect of the high resolution cutoff on SIRAS/SIR phasing. The indexed patterns of 10,792 native and 10,000 Hg derivative LRE crystals were used for this calculation. The color and symbol scheme are the same as in Fig. 1. It should be noted that the phasing was successful with lower resolution data when more patterns were used. The figure was prepared using R^36^ with ggplot2 package^37^.

With the indexed patterns of 10,792 native and 10,000 Hg derivative crystals, both SIRAS and SIR method needed higher resolution data than 1.7 or 1.8 Å (Fig. 5). However, SIRAS phasing is more robust to variation in the employed high-resolution cutoff than the SIR case.

### Quality of the anomalous signals

**Figure 6:**
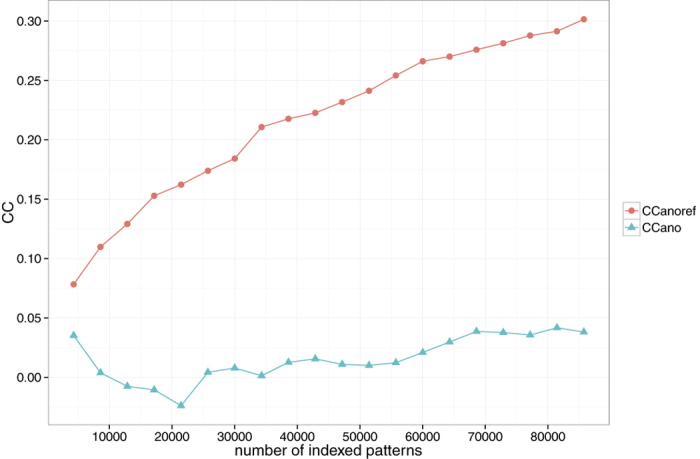
CC_ano_ and CC_anoref_ as a function of pattern numbers used for Monte-Carlo integration. CC_ano_ was calculated using two data sets grouped randomly. CC_anoref_ is defined as in the main text. The figure was prepared using R^36^ with ggplot2 package^37^.

**Figure 7:**
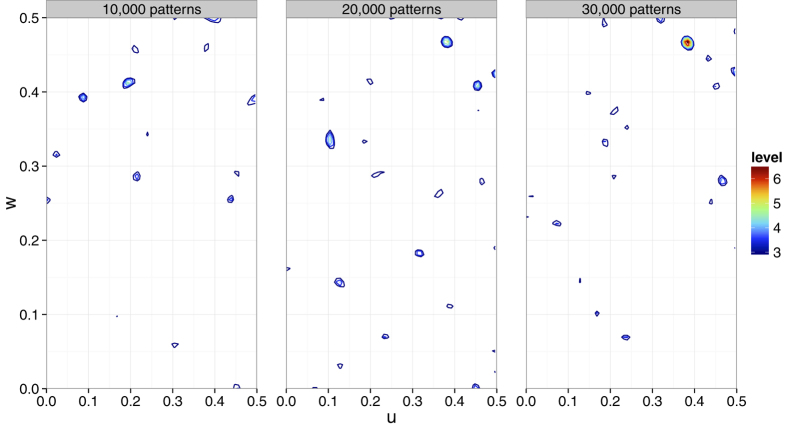
Anomalous difference Patterson maps. The *v* = 1/2 Harker section is calculated using different numbers of indexed Hg derivative patterns. When 10,000 patterns are used, a peak of 3.2*σ* is observed at the Hg position. There are, however, other peaks higher than this in the Harker section. When 20,000 and 30,000 patterns are used, Hg peaks with heights of 5.0*σ* and 6.9*σ*, respectively, are observed and they are the highest peaks in the sections. The Patterson map was calculated using the CCTBX functionality^38^. The figure was prepared using R^36^ with ggplot2 package^37^.

SFX data analysis using CrystFEL relies on the Monte-Carlo integration of Bragg reflections that partially intercept the Ewald sphere^20^,^21^. This statistical extrapolation process favors a greater number of indexed diffraction patterns for better accuracy in the measurements of reflection intensities. The correlation coefficient (CC_calc_) between *I* and 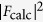 improved as more diffraction patterns were used (Supplementary Fig. 2). We compared the correlation in anomalous difference, CC_ano_, of two randomly divided data sets by changing the number of diffraction patterns. This provides a measure for the quality of the anomalous signal with respect to the number of diffraction patterns. The analysis showed that the anomalous signals were very weak; CC_ano_ was −0.066 using 10,000 patterns and remained relatively low, at 0.038 even with ~85,000 patterns. This explains why SAD phasing was unsuccessful. For more accurate analysis of anomalous signals, we introduced a new correlation coefficient termed CC_anoref_, which measures the consistency between *I*^(+)^ − *I*^(−)^ and 
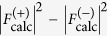
. Here 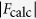 is the structure factor calculated from the LRE Hg-bound model. The model was refined against the derivative data of 10,000 patterns with the theoretical anomalous scattering contributions of the Hg atoms incorporated into the refinement. The noted monotonic increase in the CC_anoref_ values for the increased number of diffraction patterns confirms the presence and enhancement of the anomalous signals (Fig. 6). This is also confirmed by the increased peak height of the anomalous difference Patterson map (Fig. 7).

### Comparison of SFX and SR data

To support the *de novo* SFX phasing using SIRAS, we carried out further experiments on the LRE crystals using synchrotron radiation (SR) at BL26B2 of SPring-8. Diffraction patterns were collected up to 1.7 Å resolution by the oscillation method at room temperature. The SR data show a good agreement with the SFX data; the correlation coefficients for the majority of resolution shells to 2.0 Å are around 0.95, supporting the good quality of the SFX data collected at SACLA (Supplementary Fig. 3). For the SR data, phases were determined by the SAD method. Model building and refinement were performed following the same protocol used for the SFX SIRAS case. The data collection and refinement statistics are summarized in Table 1. When the refined SR structure was compared with the SFX structure, the main-chain atoms were superposed with root-mean square deviation (r.m.s.d.) of 0.21 Å. It confirmed that the structure revealed by *de novo* structure determination using SFX data is consistent with that determined by SAD phasing.

## Discussion

In this research, we demonstrated that the SIRAS method was successful for SFX *de novo* phasing with the indexed patterns of 10,792 native and 10,000 Hg derivative crystals whereas the SAD method was not successful even with 85,747 derivative crystals. The advantage of SIRAS over SAD phasing, is attained from the presence of large isomorphous differences (≳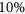 contribution to 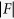) in addition to anomalous differences. The SAD method is contingent on the accurate measurement of the anomalous differences which are small (≲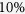 to 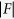).

For the application of the SIRAS method it is necessary to prepare both native and isomorphous derivative crystals. SIRAS phasing employed here benefited from both good isomorphism between crystals and the availability of high resolution data. Although non-isomorphism could be an issue, there is a reasonable chance of successful phasing by collecting data from both native and derivative crystals. We believe that SIRAS is a practical alternative for *de novo* structure determination by SFX requiring significantly less data and crystals. Use of the heavy atoms with absorption edges in high energy (near 12.4 keV) is beneficial because it enables high resolution data collection with the fixed detector dimensions.

A clear peak for a single Hg atom per asymmetric unit was observed in the anomalous difference Patterson map with 20,000 or more patterns (Fig. 7) and SHELXD was able to locate the Hg atom correctly. More patterns would be required to obtain accurate phases by SAD for modeling than for just the heavy atom position search^7^.

We expect a better data processing algorithm than the Monte-Carlo integration to facilitate efficient structure determination. Recently new developments that employ the partiality correction and the post-refinement techniques are reported^22^,^23^,^24^, which reduced the necessary amount of data. Further development in data processing method will enable efficient *de novo* phasing by SFX including SIRAS and SAD.

## Methods

### Cloning, expression, and purification of LRE

The open reading frame of LRE from *Photinus pyralis* was cloned into pET15b (Novagen) expression vector between *Nde*I and *Bam*HI sites to obtain pLRE-pET15, which enabled expression of LRE with a N-terminal cleavable 6His-tag.

*Escherichia coli* BL21(DE3) cells transformed with pLRE-pET15 plasmid were grown to an OD_600_ of 0.6 in 2 L TB medium (1.2% peptone, 2.4% yeast extract, 72 mM K_2_HPO_4_, 17 mM KH_2_PO_4_ and 0.4% glycerol) at 37 °C with shaking at 180 rpm. The protein expression of 6His-LRE was induced by adding IPTG to the culture to a final concentration of 0.2 mM and the incubation were continued at 18 °C for 16 hours with shaking at 160 rpm. A cell pellet of 25 g (wet weight) was obtained from 1 L TB medium at the end of the culture.

Purification of LRE was performed either on ice or at 4 °C. The cell pellet (50 g) was suspended in 150 mL buffer A (50 mM Tris-HCl pH 7.5, 0.1 M NaCl, 10% glycerol) containing 1 mM PMSF and the cOmplete Protease Inhibitor Cocktail (Roche) and lysed with an Emulsiflex-C3 high-pressure homogenizer (Avestin) and a Branson 250 sonicator. The clear lysate obtained from centrifugation at 25,000 × *g* for 20 min was incubated with 20 mL cOmplete His-Tag Purification Resin (Roche) for 2 hours with shaking. The resin bound with 6His-LRE was washed on an open column with 300 mL buffer A and 6His-LRE was eluted with buffer A containing 300 mM imidazole. The fractions containing 6His-LRE were pooled and the 6His-tag was cleaved by incubation with Thrombin (20U for 1 mg of 6His-LRE) at 4 °C overnight. Tag-cleaved LRE was dialyzed against buffer B (10 mM HEPES pH 7.5, 0.1 M NaCl, 10% glycerol) and concentrated to 27 mg/mL. The yield of LRE was 100 mg per 1 L TB medium.

### Crystallization and Hg-derivative preparation

Micro crystals for XFEL data collection were generated by mixing purified LRE solution (27 mg/mL LRE, 10 mM HEPES pH 7.5, 0.1 M NaCl, 10% glycerol) and precipitant solution (35% PEG3350, 10% MPD, 0.1 M HEPES pH 7.5, 0.2 M MgCl_2_) at ratios between 1:2 and 1:1.6 followed by incubation at 4 °C overnight. Micro crystals were collected by centrifugation at 1,000 × *g* for 5 min and stored in the stock solution (31.5% PEG3350, 10% MPD, 5% glycerol, 0.1 M HEPES pH 7.5, 0.2 M MgCl_2_, 0.1 M NaCl). The microseeding technique was carried out for the Hg-derivative crystals. Micro crystals containing Hg-derivative LRE were obtained by soaking native micro crystals in the stock solution containing 1 mM HgO for 6 days and then back-soaked in the stock solution for 1 hour. The micro crystals were mixed with a grease matrix^25^ before SFX data collection.

Crystals for the SR data collection were obtained by the sitting drop vapor diffusion method. 1 *μ*L of the purified LRE solution was mixed with an equal volume of reservoir solution (30.6% PEG3350, 10% MPD, 0.1 M MOPS pH 7.0, 0.2 M MgCl_2_) to form a sitting drop. The drop was then equilibrated over 600 *μ*L of the reservoir solution at 20 °C. Hg-derivative crystals were obtained by soaking the native crystals in the stock solution containing 1 mM HgO for 6 days and then back-soaked in the stock solution for 5 days. A single crystal in the stock solution was mixed with the grease^25^ using Hampton 18 mm Mounted CryoLoop (Hampton) before data collection.

### XFEL experiment

The SFX experiment was performed at BL3 of SACLA. Photon energy was tuned to 12.6 keV (0.984 Å), at which energy Hg shows an anomalous scattering contribution 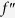 of 9.75*e*. The pulse duration was <10 fs, and the repetition rate was 30 Hz. The rod-shaped micro crystals with a width of 2–5 *μ*m and a length of 10–30 *μ*m were filtered using a mesh with pore size of 30 *μ*m then mixed with the grease^25^. The mixture was extruded from the syringe injector system installed in a DAPHNIS chamber in a moist helium environment at room temperature. The flow rate was 0.48–0.50 *μ*L/min and the inner diameter of the needle was 110 *μ*m. The diffraction patterns were collected using the multi-port CCD (MPCCD) detector with the short working distance (SWD) octal sensor arrangement^26^.

### SFX data processing

Prior to data processing by CrystFEL, images were discarded if the maximum value of low resolution area (~3.8 Å) did not exceed 5,000 ADU. Diffraction images were indexed and integrated by CrystFEL version 0.5.3a. Indexing was performed using DirAx version 1.16^27^, MOSFLM version 7.2.0^28^, and XDS version Jan 10, 2014^29^ in that order, and the first successful indexing result was used for integration. The latest feature of MOSFLM, which utilizes prior unit cell information for indexing (prior-cell option), was used and increased the indexing rate. After sample exchange the camera length was refined which resulted in higher indexing rates^30^. With the optimized camera lengths, the systematic fluctuations of the unit cell dimensions were not observed and the averaged unit cell dimensions were used for downstream analyses. While collecting the Hg-derivative data, we used a low-angle absorber to prevent the high peak intensity from strong reflections surpassing the maximum acceptable value of each detector pixel (~250,000 ADU). The absorber was made of 300 *μ*m Aluminum (36.36% transmittance at 12.6 keV) and designed to cover reflections with lower resolution than 3.8 Å. Both absorber radius and center position were determined by inspecting diffraction images on the detector. Before Monte-Carlo integration, the integrated intensities and measurement errors of the spots in the absorber region were corrected by the theoretical transmission factor. The angular dependence was not taken into account.

### SIRAS phasing and refinement

The initial phases were determined and improved by SHELXC, D, E (versions 2013/2, 2013/1, 2014/2, respectively) using the auto-tracing feature^14^. The asymmetric unit was assumed to contain one LRE molecule with a solvent content of 44%. LRE has only one Cys residue and, therefore, one Hg site was expected. Initial model building with iterative refinement by REFMAC version 5.7.0029^16^ was performed by ARP/wARP version 7.3 auto_tracing.sh^15^. Manual model rebuilding with Coot version 0.8-pre^17^ and refinement with TLS parameters using phenix.refine version 1.9^18^ were repeated. In the final refinement cycle, the automatic optimization of target function weight to balance the restraint and the X-ray terms was performed. The stereochemical qualities of the final refined model were analyzed with Phenix including MolProbity analysis^19^. The model was also refined against the derivative data. Tabulated values of Hg atomic form factors, 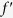 and 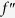, were used^31^. The refinement and validation of the Hg derivative structure were performed in the same way as the native structure.

The CC between the electron density map given by SHELXE and the map derived from the refined structure was calculated using phenix.get_cc_mtz_pdb^32^.

### SAD phasing using SR data

The diffraction dataset was collected using MX225 CCD detector (Rayonix, Evanston, IL, USA) on BL26B2, SPring-8 (Harima, Japan) at room temperature. The crystal size was 200 × 400 × 50 *μ*m^3^ and the dataset was collected using five irradiation points with step size of 35.7 *μ*m and beam size of 80 × 90 *μ*m (FWHM). The total absorbed dose for each point (36 images) was 58.7 kGy (given by RADDOSE version 2^33^,^34^). The diffraction data were indexed, integrated, and scaled by XDS^29^ at 1.7 Å resolution. The test set for *R*_free_ evaluation was transferred from the SACLA data. Initial phases were determined by SHELXC, D, E using the auto-tracing feature^14^. The initial model was automatically built by ARP/wARP^15^. A careful manual model rebuilding using Coot^17^ following the automated refinement using phenix.refine^18^ was repeated. In the final refinement cycles, the automatic weight optimization was performed. The stereochemical qualities of the final model were analyzed with Phenix including MolProbity analysis^19^. The superposition to the SFX structure was performed using LSQKAB version 6.3^35^.

## Additional Information

**Accession codes**: The coordinates and experimental data have been deposited at the Protein Data Bank (PDB) with codes 5D9B (SFX native), 5D9C (SFX Hg derivative), 5D9D (SPring-8 Hg derivative). The raw diffraction images will be available at CXIDB (http://cxidb.org) with CXIDB ID 31.

**How to cite this article**: Yamashita, K. *et al*. An isomorphous replacement method for efficient *de novo* phasing for serial femtosecond crystallography. *Sci. Rep*. **5**, 14017; doi: 10.1038/srep14017 (2015).

## Supplementary Material

Supplementary Information

## Figures and Tables

**Table 1:** Data collection and refinement statistics.

	XFEL (SIRAS)		SR (SAD)
	Native	Hg-derivative	Hg-derivative
Beamline	SACLA BL3		SPring-8 BL26B2
Wavelength (Å)	0.981	0.984	0.9839
Beam energy* or photon flux	30.0 *μ*J/pulse	86.4 *μ*J/pulse	2.0 × 10^10^ phs/s
X-ray detector	MPCCD (short working distance octal)		MX225
Space group	*P*2_1_2_1_2_1_	*P*2_1_2_1_2_1_	*P*2_1_2_1_2_1_
Unit cell (*a*, *b*, *c*; Å)	48.2, 77.6, 84.8	48.1, 77.5, 84.8	48.11, 77.18, 84.92
Resolution range† (Å)	10–1.50 (1.56–1.50)	25–1.60 (1.66–1.60)	40–1.70 (1.80–1.70)
Completeness† (%)	100 (100)	100 (100)	99.8 (99.3)
SFX multiplicity†	222.2 (196)	106.2 (44)	n/a
Redundancy†	n/a	n/a	3.9 (3.8)
No. crystals	10,792	10,000	1
*R*_split_†,§	0.2727 (1.671)	0.3727 (5.772)	n/a
*R*_meas_†,¶	n/a	n/a	0.101 (0.728)
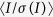 †,‡	2.56 (0.63)	1.82 (0.19)	10.58 (2.15)
CC_1/2_†,#	0.893 (0.1988)	0.835 (0.0279)	0.997 (0.696)
CC_ano_**	n/a	−0.066	0.16
Refinement			
*R*_work,_ *R*_free_	0.1845, 0.2318	0.2021, 0.2354	0.1444, 0.1776
No. atoms (mean *B*-factor)			
protein	2,427 (27.0)	2,403 (27.8)	2,456 (22.6)
water	188 (39.9)	164 (35.6)	158 (35.0)
ligand/ion	9 (32.6)	9 (30.6)	9 (44.6)
Hg	n/a	2 (25.6)	3 (21.8)
r.m.s. deviation from ideal			
bond lengths (Å)	0.011	0.014	0.019
bond angles (°)	1.346	1.452	1.778
Ramachandran plot			
Favored (%)	97.76	95.78	95.25
Allowed (%)	2.24	4.22	4.43
Outlier (%)	0	0	0.32

^*^XFEL beam energy calculated from the reflectivity or the transmittance of the components between the beam monitor and the sample position (attenuator, KB-mirrors, Be windows, and air path).

^†^Values in parenthesis are for the highest resolution shell.

^‡^Note that *σ*(*I*) estimation method is different between CrystFEL and XDS and they cannot be compared.

^§^
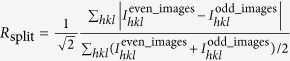
12.

^¶^
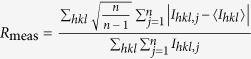
40.

^#^CC_1/2_ is a correlation coefficient of intensities between randomly-halved datasets^41^.

^**^CC_ano_ is a correlation coefficient of anomalous intensity differences between randomly-halved datasets.
