# Supplementary material for: An isomorphous replacement method for efficient de novo phasing for serial femtosecond crystallography
Source: Sci Rep. 2015 Sep 11;5:14017. doi: 10.1038/srep14017 (PMC4566134; doi:10.1038/srep14017)
Supplement: Supplementary Information [file srep14017-s1.pdf]

An isomorphous replacement method for efficient *de novo* phasing for serial femtosecond crystallography

Keitaro Yamashita, Dongqing Pan, Tomohiko Okuda, Michihiro Sugahara, Atsushi Kodan, Tomohiro Yamaguchi, Tomohiro Murai, Keiko Gomi, Naoki Kajiyama, Eiichi Mizohata, Mamoru Suzuki, Eriko Nango, Kensuke Tono, Yasumasa Joti, Takashi Kameshima, Jaehyun Park, Changyong Song, Takaki Hatsui, Makina Yabashi, So Iwata, Hiroaki Kato, Hideo Ago, Masaki Yamamoto, Toru Nakatsu*

Supplementary Figures

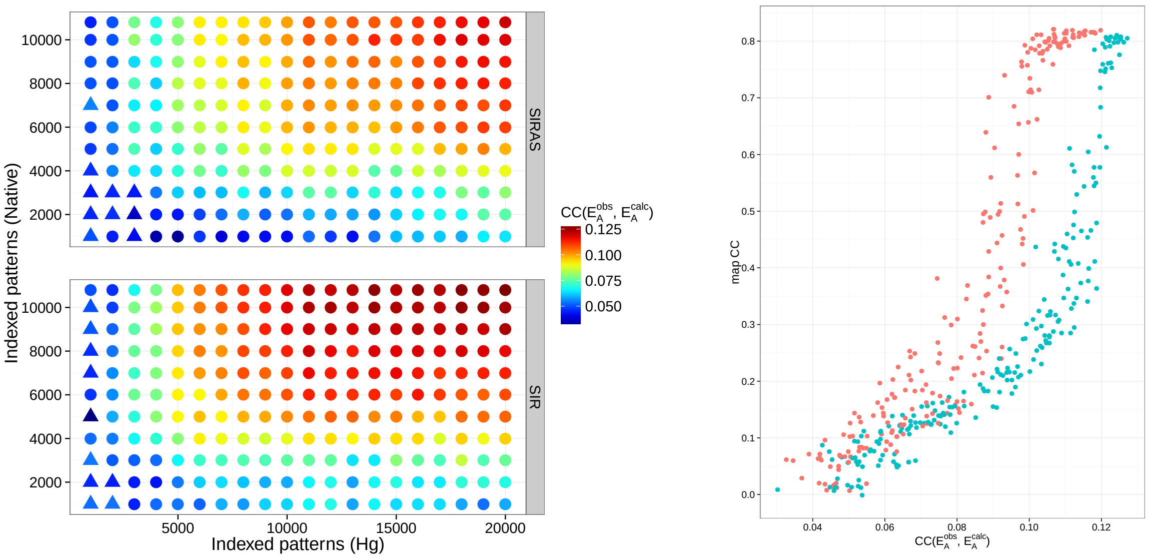

(a) (b)

Supplementary Figure 1: **Heavy atom site correctness, accuracy of substructure structure amplitudes, and its relationship to phasing.** (a) Correlation coefficient between E_A^{obs} from estimated substructure structure amplitudes and E_A^{calc} from heavy atoms located by SHELXD [1] is plotted as functions of the numbers of the native and the derivative indexed patterns. This CC(E_A^{obs}, E_A^{calc}) was calculated by SHELXE [2]. Data points are represented as circles if a site located by SHELXD is within 0.5 Å of the correct site identified by ANODE [3]; otherwise triangles. (b) The relationship between CC(E_A^{obs}, E_A^{calc}) and the map CC as in Fig. 1 in main text. Higher CC(E_A^{obs}, E_A^{calc}) has higher chance of successful phasing for each method. The figures were prepared using R [4] with ggplot2 package [5].

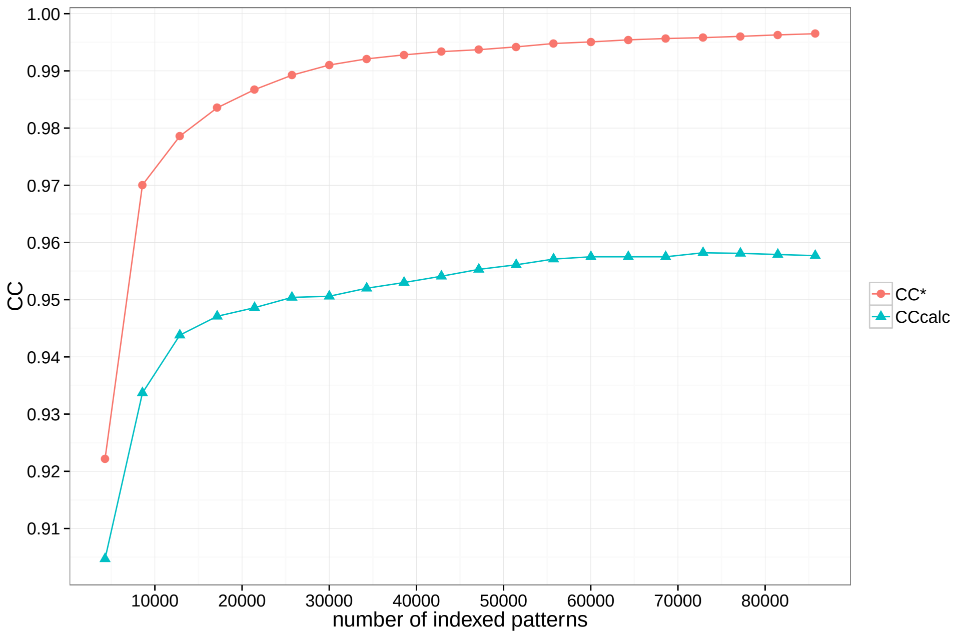

Supplementary Figure 2: **CC_{calc} and CC^* as a function of pattern numbers used for Monte-Carlo integration.** CC_{calc} was calculated using F_{calc}, the calculated structure factor of the LRE Hg-bound model refined against the derivative data of 10,000 patterns. CC^* is an estimate for CC with true signal and calculated from $CC_{1/2}$ $\left(CC^* = \sqrt{2CC_{1/2}/(1 + CC_{1/2})}\right)$ [6]. The figure was prepared using R [4] with the ggplot2 package [5].

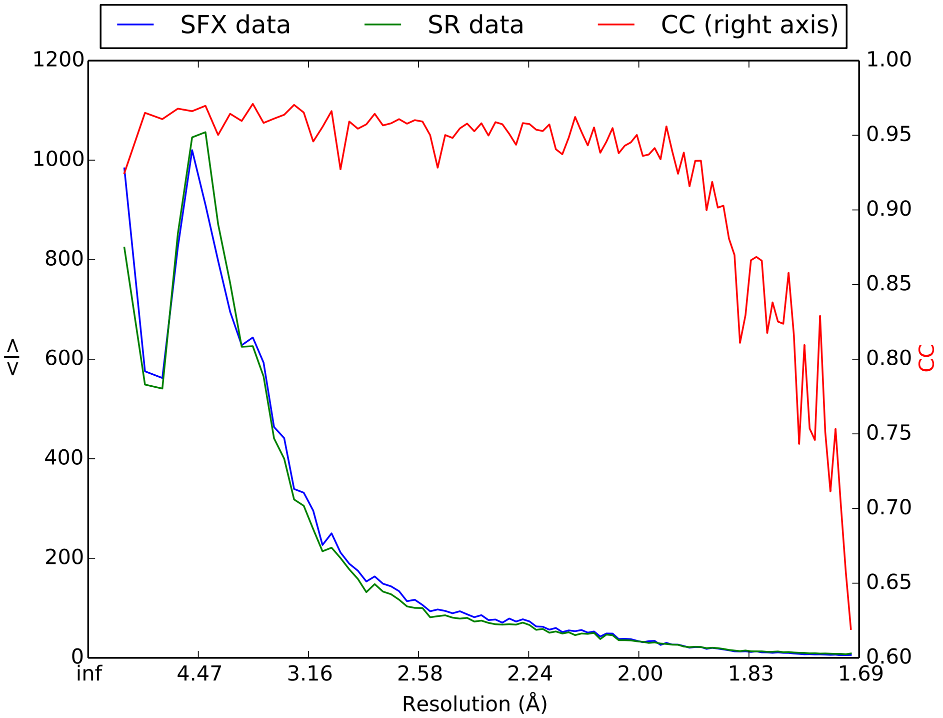

Supplementary Figure 3: **Comparison of SFX and SR data.** Averaged intensities in each resolution bin of the SFX data using $\sim 30,000$ patterns (blue line) and the SR data (green line) of the Hg derivative LRE crystal are shown. They are scaled with a linear scale factor and B, which were calculated with CCTBX functionality [7]. A good agreement suggests that the low-angle absorber used in SFX data collection did not deteriorate the data and mitigated a potential detector saturation problem. The figure was prepared using Matplotlib library [8].

[1] Schneider, T. R. & Sheldrick, G. M. Substructure solution with *SHELXD*. *Acta Crystallographica Section D* **58**, 1772–1779 (2002).

[2] Sheldrick, G. M. Macromolecular phasing with SHELXE. *Zeitschrift für Kristallographie* **217**, 644–650 (2002).

[3] Thorn, A. & Sheldrick, G. M. *ANODE*: anomalous and heavy-atom density calculation. *Journal of Applied Crystallography* **44**, 1285–1287 (2011).

[4] R Development Core Team. *R: A Language and Environment for Statistical Computing.* R Foundation for Statistical Computing, Vienna, Austria (2008).

[5] Wickham, H. *ggplot2: elegant graphics for data analysis* (Springer New York, 2009).

[6] Karplus, P. A. & Diederichs, K. Linking Crystallographic Model and Data Quality. *Science* **336**, 1030–1033 (2012).

[7] Grosse-Kunstleve, R. W., Sauter, N. K., Moriarty, N. W. & Adams, P. D. The *Computational Crystallography Toolbox*: crystallographic algorithms in a reusable software framework. *Journal of Applied Crystallography* **35**, 126–136 (2002).

[8] Hunter, J. D. Matplotlib: A 2D graphics environment. *Computing In Science & Engineering* **9**, 90–95 (2007).
